# Read like a man: comparing narratives of masculinity in adolescent literature

**DOI:** 10.3389/fsoc.2024.1329041

**Published:** 2024-03-13

**Authors:** Francisco Hernandez, Michael Macaluso

**Affiliations:** ^1^St. Philip & St. Augustine Catholic Academy, Dallas, TX, United States; ^2^University of Notre Dame, Notre Dame, IN, United States

**Keywords:** literary analysis, adolescence, literary (anti)canon, masculinity, education, literature, gender, young adult literature (YAL)

## Abstract

Recent movements like #MeToo and #TimesUp have surfaced and challenged ideas about masculinity in popular conversations. In particular, these ideas have centered around “toxic masculinity”—a version of masculinity that reflects stereotyped, dated, and even dangerous expectations for manhood. This notion of masculinity can be reinforced in a number of ways, especially through pop culture, where it runs the risk of becoming commonly accepted or normalized. This study evaluates the narratives of masculinity in three different novels that are marketed toward high school-aged students in the United States: *Lord of the Flies*; *Gabi, A Girl in Pieces*; and *Aristotle and Dante Discover the Secrets of the Universe*. Using a critical literacy lens, this study considers the symbols, behaviors, expectations, and meanings given to masculinity in and through each novel and considers the implications of this analysis for adolescent readership inside and outside of schooling. The study concludes that the more contemporary novels showcase a range of masculine portrayals, including positive, affirming versions of masculinity, compared to a more singular and pessimistic one found in a novel traditionally used in schools. Thus, the study concludes that formal schooling may be an important way to address and disrupt unhealthy versions of masculinity.

## Introduction

The implications and reverberations from the #MeToo and #TimesUp movements have engaged us in critical conversations around the idea of masculinity. From pop culture to politics, these movements forced individuals to examine how men in positions of unchecked power and privilege, throughout Hollywood and other industries, sexually assaulted women without consequence. These movements, though aimed to help survivors—largely women—of sexual violence, have cast a wider net in recent years to identify the systematic causes and ideologies of harassment. Indeed, a quick glance at the headlines on any given day reveals a stark reality: continued stories and instances from #MeToo survivors, political degradation on both sides, instances of police brutality, and references to the rise in adolescent suicide rates attributed to bullying. As such, many have suggested that these issues stem from a larger sociocultural problem of “toxic masculinity,” a value system incorporating structures, beliefs, and expectations around gender and power.

Toxic masculinity (Connell, 2005; Clemens, 2017; De Boise, 2019), in brief, captures the traditional cultural and societal stereotypes of masculinity and manhood. The term is not a new one, as it has long signified traits like aggression, violence, status, and a desire for control and the oppression of others (especially women), and it has even become a popular term in cultural circles. For example, in response to toxic masculinity, the shaving company Gillette launched a campaign, #TheBestMenCanBe, drawing on their tagline of “the best a man can get,” to celebrate and help men achieve their personal best. In doing so, their advertisement campaign brought critical attention to the ways in which society and media have contributed to sexual assault crimes. The commercial shows examples of behaviors that contribute to the problem of toxic masculinity: catcalling, demeaning women in professional settings, men happily being publicly promiscuous, and boys fighting and bullying each other. The short film proposes that media artifacts have normalized these behaviors in men without consequences. As a result, it denounces the “boys will be boys” mantra (Clemens, 2017) for excusing these problematic behaviors. The end of the video shows clips of men intervening and stopping these problematic behaviors. It calls men to act better, because they can act better. This ad was one of the first media artifacts to garner attention for specifically showing examples of toxic masculinity and insisting that others acknowledge their role in stopping toxicity in their daily lives.

This conversation in popular media begs another one: outside of popular culture, where, when, and how might toxic masculinity be disrupted for young men and women? As current educators, we argue that if schools are structures of social and cultural reproduction—institutions with curriculum, pedagogy, and community capable of socially *reproducing* toxic masculinity—then they must also be sites capable of critically *disrupting* and actively *engaging* with oppressive ideologies like toxic masculinity. We argue that if we are to engage in the conversation of toxic masculinity, then we must find a place to do so *within* schools. More specifically, we believe that teaching with a lens of toxic masculinity in English Language Arts classrooms might help to dispel and disrupt dangerous notions and ideologies of toxic masculinity.

## Critical engagement in schools

In the spirit of this critical engagement, educators have begun to critically engage with established high school canonical texts as curricular sites of social reproduction. They have reconsidered highly esteemed, usually unquestioned novels commonly taught in high school. These educators, using a lens of critical literacy, engage in the more implied narratives written in these texts, aiming “to draw attention to implicit ideologies of texts and textual practices by examining issues of power, normativity, and representation, as well as facilitating opportunities for equity-oriented sociopolitical action” (Borsheim-Black et al., 2014, p. 123). For example, Macaluso’s research on the typical high school text *To Kill a Mockingbird*, challenges its traditionally accepted anti-racist themes for its subtle racist underpinnings (Macaluso, 2017). While Atticus is praised for his above-the-law morals for defending Tom Robinson against the social esteem of his peers, we should consider how this continual reading promotes a White-Savior narrative, normalizing that African Americans have little to no agency. This study is done not only to reconsider the unquestioned texts but also to teach students these literacy skills: consume literature, engage with it and the ideologies of the text, and reconsider their sociopolitical implications. Steiss (2020) has done similar work in teaching Homeric canonical texts more critically, instead of completely replacing them. He argues that the Homeric texts offer students the opportunity to understand and question narratives in a productive way, noting, “After reading each episode and asking questions about whose perspectives are valued and whose are silenced, many students noted an absence of the women’s perspectives and much evidence that they were actually the victims in these encounters” (Steiss, 2020, p. 436). His approach of not completely replacing canonical novels but offering the text as an opportunity for critical insight is a balanced approach, one that is necessary given the fact that texts like *The Odyssey* (and *To Kill a Mockingbird*) are already in the social consciousness of American culture. Rather than remove them completely from the classroom, a critical literacy approach allows these texts to be opened up to further questioning and contemporary critique.

Current young adult literature (YA), on the other hand, tends to more explicitly engage in contemporary critical discussions, questioning and disrupting normative values. Additionally, contemporary YA can be culturally representative and, thus, more relevant to student’s lived experiences, highlighting cultural knowledge and contemporary struggles. As a result, some educators have made YA more available in their classrooms: as options for independent reading, through small group literature circles, or by pairing it alongside a canonical text for classroom study. Regardless of the method, thematic conversations around the canon and YA could engage students in larger critical thought processes, contextualizing the author’s *and* reader’s beliefs, values, and identities. Even if students do not read YA as part of classroom study, they may be more likely to pick up YA texts outside of the classroom.

In this article, we take up the contemporary topic of masculinity, particularly toxic masculinity, in reading across two recently published, highly acclaimed YA novels—*Aristotle and Dante Discover the Secrets of the Universe* (*A&D*) by Benjamin Alire Sáenz; *Gabi, A Girl in Pieces* (*Gabi*) by Isabel Quintero—and one more traditional, canonical novel that has long been taught in schools: *Lord of the Flies* (*LOF*) by William Golding. *LOF* has been and continues to be widely taught in middle and high schools in the United States, but not necessarily in *Gabi* and *A&D*. We are not necessarily advocating for the use of *A&D* and *Gabi* in classrooms, as both contain content that teachers may deem too mature or explicit for classroom inclusion, but then again, so does *LOF* in the form of young children brutally murdering other children. That said, teachers must use their discretion and knowledge of their students and context to determine what will work for them. We chose *A&D* and *Gabi* for this study a number of reasons—both have been written recently, feature diverse characters and contexts, have won a number of literary awards, rank highly on user-reviewed book sites like Goodreads.com, and both were named on the “Best Books” list for their respective year from *School Library Journal*, a publication specifically geared toward recommendations and reviews for schools, school librarians, and classroom teachers. During our discussions of these novels, we kept coming back to *LOF*, a text we both read in school, noting that all three texts ground masculinity as one focal point.

Using a critical literacy lens to our content analysis (Krippendorff, 2004), we noted common themes or motifs regarding masculinity across these three books. We first considered the ways in which *LOF* constructs a narrative of masculinity and then compared that narrative to the ways in which the YA texts might critically disrupt those narratives in *LOF*. We highlight the narratives, images, and conclusions these books make on masculinity as both a gender construct and a philosophical claim. The following questions guided our analysis:

How do contemporary narratives about masculinity, as seen in the two YA texts, compare to those in *LOF*, a more traditional text found in classrooms?

What depictions might characterize a toxic or antagonistic male?

Is any version of masculinity, across the three texts, valued and/or valorized?

What are the social implications of these depictions of masculinity as represented in texts assigned to schools?

This study was inspired by our mutual interests in masculinity, recognizing the ways in which behaviors of, about, and for masculinity were reified early in our lives through family, friends, media, religious beliefs, and cultural expectations. We first explored these ideas when Hernandez was a student in Macaluso’s undergraduate literature seminar several years ago. Since then, we have continued the conversation through an independent seminar, and now, as a current English teacher and English teacher educator. We agree that books—like movies, songs, TV shows, social media, and other cultural artifacts—can play an important role in affirming and/or challenging stereotyped notions of masculinity.

## Canon

*Lord of the Flies*

*Lord of the Flies* is one of the most popular books to teach in high school literature classes (Macaluso, 2016). Educators and students will find a plethora of symbolism embedded in multiple layers of the castaway story: pig heads, war paint, glasses, the conch, the beastie, fruits in a garden, and repeated “*sucks to your asmarr*” remark. Additionally, the novel offers the opportunity for students to consider ethical or unethical actions of each character and their affiliation with their own agendas. *Lord of the Flies* is an established canonical text of high school literature because it provides a variety of avenues and interpretations.

The novel explores the concept of masculinity through its characterization and conflict. More specifically, complicating masculine models is integral to understanding conflict between the characters in the novel. If readers do not read *LOF* through a lens of masculinity, they lose the author’s original purposes in the conception of the novel itself. Interviews with Golding report that *LOF* was inspired by his personal reflections on the portrayal of masculinity in the British classic written by R.M. Ballantyne *Coral Island*. Samuel Hynes (Baker, 1988) cites an interview where Golding discusses masculinity in the context of the castaway genre in British literature,

What I’m saying to myself is “don’t be such a fool, you remember when you were a boy, a small boy, how you lived on that island with Ralph and Jack and Peterkin.” …I said to myself finally, “Now you are grown up, you are adult; it’s taken you a long time to become adult, but now you’ve got there you can see that people are not like that; they would not behave like that if they were God-fearing English gentlemen … There, savagery would not be found in natives on an island. As like as not they would find savages who were kindly and uncomplicated and that the devil would rise out of the intellectual complications of the three white men on the island itself (Baker, 1988, p. 16).

Golding’s comments critique the previously established castaway texts he encountered as a child, and he critiques the portrayal of British men in the setting of a castaway, stating their incorrect premise of what is “savage.” Previous canon established British men as gentlemen, heroes, and civilized in these stories, while portraying the non-white, non-British characters as terrors and savages of all that is good, holy, and seemingly. Hynes calls this the “Coral Island attitude” in reference to the castaway tale *Coral Island* (Baker, 1988).

Countering the “Coral Island Attitude,” Golding argued that the real “savages” are not the fictional indigenous groups that represent indigenous peoples; rather, the real source of uncivilized actions and moral evil would come from the “three white men” castaway on the island. At the very end, after all of the events have transpired on the island, the naval officer, surprised by Ralph’s disheveled looks and the burning island, states ironically,

“I should have thought that a pack of British boys—you’re all British, aren’t you?—would have been able to put up a better show than that—I mean—”

“It was like that at first,” said Ralph,” before things—”

He stopped.

“We were together then—”. The officer nodded helpfully.

“I know. Jolly good show. Like the Coral Island.”

…Ralph wept for the end of innocence, the darkness of man’s heart, and the fall through the air of the true wise friend called Piggy.” (Golding, 1962, p. 242)

Characters in the novel, and readers alike, come to the realization that the *Coral Island* depiction of reason and moral goodness is not a conclusive narrative for the British boys. *LOF* critically disrupts the ideology that British masculinity is intrinsically innocent, Christian, and superior. In his new goal for realism, *LOF* creates a new masculinity narrative, one that Golding claims to be more “realistic” and grounded in violence, aggression, and even sexually predatory. In this section, we explore some of these possible narratives.

### Piggy: feminized boy?

Piggy stands out from the rest of the boys in the novel in a number of ways. While most of the main characters identify with their fathers, Piggy identifies with his Auntie because his father is notably passed away (p. 19). Furthermore, his asthma and glasses put him in a physical disadvantage compared to the rest of the able-bodied boys, who can use physical strength to command others (p. 19). From the perspective of the boys on the island, Piggy does not live up to their masculine conception and expectation; therefore, he is generally mistreated, ostracized, and seen as different.

In addition to Piggy’s unique characterization in contrast with the boys, Piggy’s lack of masculinity invites comparisons to another female on the island, the mother pig. Both share physical and active character traits. For example, “[Piggy] was the only boy on the island whose hair never seemed to grow … [his] hair still lay in wisps over his head as though baldness were his natural state” (p. 81). Piggy’s hair resembles that of a domesticated pig, covered in wispy hair and baldness. Additionally, Piggy turns pink when shamed by the boys, sharing in the color of the pink pigs (p. 19), and Piggy is described large and “fat,” similar to pigs (p. 81).

In action, Piggy, like the Sow, cares for the young on the island. Just as the Sow cares for her piglets (p. 166), Piggy is the first to look out for the concerns of the little ones and realize that one of them went missing on the first day (p. 59). These actions and concerns contrast with the rest of the boys and their initial concerns for adventure and fun (p. 55). The text also alludes their “outcast” status on the island. For the pig, the text notes, “A little apart from the rest, sunk in deep maternal bliss, lay the largest sow of the lot. She was black and pink; and the great bladder of her belly was fringed with a row of piglets that slept or borrowed and squeaked” (p. 166). These details not only define Piggy and the Sow from the other boys, but they also seem to more intentionally align Piggy with representations of traditional femininity (like nurturing motherhood) as further indicative of his lacking masculinity.

It comes as no surprise, then, that Piggy and the Sow reach similar fates. The boys kill the Sow in a mad chase with no rational thought, “the sow staggered her way ahead of them, bleeding and mad, and the hunters followed, wedded to her in lust, excited by the long chase and the dropped blood” (p. 167). Note the imagery of a sexual conquest in the Sow. Similar to the Sow, Piggy is killed by Roger in a “sense of delirious abandonment” (p. 222). The novel directly compares Piggy’s body to that of the Sow: “Piggy’s arms and legs twitched a bit, like a pig’s after it has been killed” (p. 223). In both cases, masculine control of the feminine, via sexual conquest, is associated with the boys of the island, and subsequently celebrated. This is a dangerous narrative to reify, however implicit, to readers of the text, as it signals assault, power, and violence over others, particularly women.

### Jack: masculinity as dominance

Contrasted to Piggy, Jack is characterized by traditionally masculine traits, and is in conflict with him constantly. Jack’s identity and actions antagonize both Piggy and the Sow. Jack’s quick leadership and actions are imbued with images of destruction and aggression toward the boys, especially Piggy. In an early confrontation, “Jack stood up as he said this, the bloodied knife in his hand. The two boys faced each other. There was the brilliant world of hunting, tactics, fierce exhilaration, skill; and there was the world of longing and baffled common-sense” (p. 89). Jack’s self-conception and course of action is completely opposite of Piggy who stands for “common-sense.” In this narrative of masculinity, *LOF* establishes a clear relationship of power; Jack is defined by a masculinity that is physically powerful, dominative, and even irrational. His masculinity seeks out to antagonize femininity, as established by his aggression and killing of the Sow and Piggy.

His masculinity is also manifest through domination of the other boys; whether it is iron-fisted control of the choir boys or setting the agenda and rituals for the same boys turned hunters. The masculinized desire for dominance is demonstrated in the younger boys on the beach as time passed on the island, suggesting that Jack’s version of masculinity is indeed the more natural version for boys and men as the boys un-civilize themselves.

### Island and society: dominance, destruction, and hopelessness?

The motif of masculine dominance is not contained on the island. The culture of civilized society may have stopped some of the boys from committing actions of aggression and dominance, but the culture, as in the world of the novel, cannot save the boys. This is made clear with commentary like, “Roger’s arm was conditioned by a civilization that knew nothing of him and was in ruins” (p. 78). The adult-world is in just as much disorder and violence as the boys’ island. As such, Golding successfully disrupts the narrative of elitist, innocent British masculinity by depicting an aggressive, dominating masculinity in Jack and the hunters. Golding depicts a new narrative in which he highlights, like contemporary movements in our age, the destructive capacity of a masculinity defined by dominance, sexual conquest, and anti-feminine sentiments.

However, Golding leaves an unsettling answer: can a new, positive, life-affirming masculinity survive realistically? Jack’s masculinity is not the only option. Ralph, Samneric, Piggy, and Simon did not function in Jack’s masculinity model of aggression and dominance. However, they barely survived or did not survive at all. The world of the novel is exaggerated and fictional, but the fates of Simon and Piggy do not leave much hope for the existence of a positive, productive version of masculinity. Additionally, although Piggy may be a boy on the island, his association with the female Sow may suggest that attributes like caring for those most vulnerable and displaying rational thought are feminine by nature.

From these conclusions and questions, *LOF* attempts a realistic but pessimistic narrative of masculinity. Masculinity and femininity are at odds with each other in agenda and values. For the educator, the *LOF* narrative of the dominating, violent, irrational masculinity in the core of the boys provides no solution to the evil nor the possibility of remediation or good. Masculinity is then strictly defined; a boy, at his natural core, is destined “to ruin.” Without deliberate disruption of these ideas, *LOF* runs the risk of reproducing and reaffirming these toxic models of masculinity.

## Young adult literature

*Gabi, A Girl in Pieces.*

### Why Gabi?

*Gabi: A Girl in Pieces*, like *LOF*, is another text filled with images, ideas, and claims about masculinity. Readers follow Gabi, a Mexican-American high schooler, and her coming-of-age story of overcoming academic, cultural, and financial obstacles. She also encounters various forms, mini-rituals, behaviors, and ideologies of masculinity in her everyday life. Over the course of the novel, she comes to understand how masculinity influences various factors of her everyday life.

In *Gabi*, there are two vehicles that introduce the story of masculinity: (1) Gabi’s romantic interests and relationships that are significant to her character arc, and (2) the cultural systems that contextualize her life, relationships, and family. Quintero distinguishes between agreeable masculine models against the machista, toxic masculinity through Gabi’s experiences with these two versions. The possibility of *positive masculinity*, as seen in the former, is significant insofar as it adds more productive answers to the masculinity conversation currently discussed.

This section analyzes the construction of masculinity narratives through Gabi’s love-interest/relationship arcs. The larger premise of the novel is unbraided: masculinity narratives and cultural narratives are intimately tied together. The end of this section compares the different masculinity narratives that *Gabi* and *LOF* offer.

### Gabi and the rules of engagement

Romantic relationships, sex, culture, and self-image are central to the novel. This opening paragraph of the novel sets up these themes and how they intertwine in Gabi’s experience of her senior year of high school:

Every time I go out with a guy, my mom says, “ojos abiertos, piernas cerradas.” Eyes open, legs closed. That’s as far as the birds and the bees talk has gone. And I don’t mind it. I don’t necessarily agree with that whole wait-until-you’re married crap though. I mean, this is America and the twenty-first century, not Mexico one hundred years ago. But of course, I can’t tell my mom that because she’ll think I’m bad.

Or worse: trying to be white (Quintero, 2014, p.7).

As stated previously, Gabi’s dating experience is the vehicle for how Quintero frames and critiques masculine norms and behaviors. This short passage, again the opening passage of the novel, offers analytic insight into Gabi as a character and the way she identifies herself in relation to her mother and societal norms. On one level, we see a clear distinction or change around sex on a generational level—Gabi is much more open to sexual encounters than her mother would like her to be, and her mother likens sexual activity with “bad” behavior. Gabi attributes her mother’s attitude to generational thinking (“this is … the twenty-first century”) and cultural and racial differences (Mexico vs. America and white vs. brown). From the start of the novel, Gabi signals that she is not those things, and thus, she does not conform to traditional (or previous generation’s) notions of femininity. We also see this non-traditional stance across her dating life and her interest in a number of boys (even at the same time) over the course of the novel. Because she is interested in so many boys—including Joshua, Martin, Eric, and Ian—readers see several examples of expressed masculine sexuality and behavior in the novel.

### Martin: positivity through emotional and sexual fulfillment

Out of all the suitors, Martin is Gabi’s favorite and most agreeable to readers. While Gabi’s other interests present some positive aspects of masculinity, they eventually fall short in some areas. For example, Ian is only interested in Gabi for sex, Eric bullies Gabi’s gay best friend, and Joshua stands up Gabi on a scheduled date night. In light of these instances, and despite their positive attributes, we posit that Martin’s attitudes and actions provide an antidote to toxic masculinity by firmly upholding Gabi’s agency, interests, sexuality, and emotional health. In essence, this is the message of the book—in the face of so much male toxicity (including her own father and another character, German, who is discussed below), there is the possibility for a clear, practical, healthy version of (non-toxic) masculinity that can be expressed through teenage boys via Martin’s characterization.

In the context of the book, sexual responsibility is valuable because irresponsibility plagues female characters with long-lasting, negative impacts. Gabi states, “I could be in the same boat as Cindy or Georgina or my mom. And that is not anywhere near where I want to be at this moment in my life. I want to go to college. I want to be free. I want to move out of this one-horse town” (p. 243). All three women she mentions have experienced challenging life-situations because of unplanned pregnancy and abusive partners, causing difficult circumstances that limit their ability to choose and succeed financially and professionally.

Martin upholds Gabi’s agency through his responsible sexuality. Martin respects Gabi’s decisions and never assumes her decisions, especially her decisions about her body. His responsibility is demonstrated when he asks for consent while kissing (p. 239) and before sexual activity during prom night (p. 247). Gabi ultimately exhibits control of her own agency by purchasing condoms. But Martin’s condom purchase is an affirmation of her agency, her desire for freedom and choices in future.

Martin’s sexuality is a free and mutual expression of attraction between Gabi and himself. This *mutual* affirmation of each other’s agency is significant because it shows that masculinity in the context of romantic relationships, sex can work in congruence with femininity and not just in conflict against it.

### German and Joshua: counters to Martin

Martin’s sexual responsibility contrasts against German’s sexual and egoist predation. German is a character who dates one of Gabi’s friends, and early on in the text, Gabi characterizes him as “one of those guys who knows he’s super hot and assumes that girls HAVE to like him” (14). Gabi’s perception of German’s lack of integrity is confirmed later, when it is revealed that he raped Gabi’s friend. When he is confronted and exposed about his crime, he replies, “Rape? Pfft. She wanted it. How could she not? All girls want this’” (260). German is a character who violates women and denies their agency, and yet his actions are written off with the familiar mantra “boys will be boys.” As a result, Gabi intentionally challenges this mindset as “a load of bullshit” (p. 229).

Joshua is another unideal suitor, as his sexual irresponsibility puts women in situations without decisions. His irresponsibility is demonstrated in Georgina’s unplanned pregnancy. Her pregnancy created an impossible situation; she must either keep her unplanned child while facing abuse from her already abusive father or violate her personal conscience by getting an abortion to avoid the abuse and life-changing consequences of having a child. Joshua left Georgina with little room for choices, nor was he present for emotional support during those decisions.

Through these different boys, Quintero constructs toxic masculinity in different forms. Both of these men violate women’s agency: German quite clearly, and Joshua more subtly. German is toxic because of his perverted sexuality and perverted sense of self; he physically and emotionally objectifies women for his pleasure and ego and never acknowledges their agency. Joshua may not be a perverted rapist, but he is sexually promiscuous and indifferent to the consequences. Similarly, indifferent to Georgina’s pregnancy dilemma, he places her in physical harm and emotional trauma.

Martin really stands out as an antithesis adolescent boy because his masculinity is characterized in such a way that it disrupts the novel’s other characters. Martin supports Gabi in her poetry work (p. 135), listens to her while she grieves her father’s death (p. 159), provides positive affirmation about her body image issues and self-deprecation (p. 170), and is sexually responsive. Quintero realistically allows for growth in positive masculinity, learning from femininity and how to support it. Martin is not perfect, but the point is that he learns. Not only does Quintero critique toxic men by giving us examples like German and Joshua, but she also gives a positive example of what masculinity should and can be.

Beyond the models, *Gabi* demonstrates how culture and society construct masculine concepts. This point is significant for its educational value; society and culture inform how men should act, and men’s behavior informs expectations in society and culture. This is seen in the excerpt below, a “boys will be boys list,” constructed by Gabi, to prove that society creates and expects toxic males:

Instructions for understanding that boys will be boys really means

1. You’re wearing that little dress tonight? Remember, boys will be boys, so be careful.
2. If you drink way too much, your body is fair game—for anyone or anyones. Boys will be boys, and you just made it easier….
3. If she is crying, that is definitely a sign that she means no. But since you are an asshole, you will not give it a second thought, so proceed. She was wearing that little dress (remember?), and boys will be boys, after all. That’s what our parents say….
4. Remember how your mother warned you that boys only want one thing from you? Well, it is not your straight A’s or your excellent drawing skills or your extensive knowledge of action films. It is the thing you have guarded (hit: it is between your legs) your whole life from everyone: your cousin who came to stay for 2 weeks, your strange uncle Tony, that teacher in the 2nd grade—they were all just boys being boys (p. 230).

Gabi identifies that this socially accepted mantra and ideology of “boys will be boys” is an excuse that perpetuates toxic behavior in men.

In addition to this list, Gabi’s reflection on Georgina’s pregnancy further elaborates the strong impact society has on the attitudes and beliefs that interpret men’s actions. Gabi writes,

Why did Georgina have to make the choices about her baby? And then live with the guilt and the fear of being found out and being labeled slut and baby killer *while Joshua Moore paraded around like nothing ever happened?* [emphasis added] Like he never had an almost-child? I mean Georgina totally helped him out too—now he does not have a responsibility and is free to go and play football or soccer or wrestle bears or whatever it is he is doing to get college scholarships. And she’s the one who’s wracked with guilt (p. 204).

The “boys will be boys” mantra intersects with Gabi’s family dynamics, not just friend dynamics. Gabi’s mother has internalized that men are hypersexual from her personal experiences, but she does not see the possibility of changing the culture. When confronted with the double standards she holds, Gabi’s mother states, “It’s different. Beto is a boy, and they cannot help it” (p. 236). Gabi’s mother clearly identifies the sexual objectification of women by hypersexual men when she states, “ya no sirve uno para nada” [after men have sex with you, men find you worthless] (p. 146). Gabi’s mom does not realize that this problematic social norm about men has been internalized in herself without questioning its dangerous implications.

Continuing with the issue of double standards and sexuality, Gabi identifies the demonization of women’s sexual desires from the machista culture. Gabi understands the ways in which culture judges and condemns female sexual desires:

[I’m] not ashamed at that. Well, a little. Because girls shouldn’t be boy crazy right? That’s what my mom always says. She says that we don’t want to be *faciles*—easy, sluts, hoes, or *ofrecidas*. And that being this way was what got Cindy in trouble, and, unless I want to follow in her footsteps, I should think twice about going out with Joshua. She says she knows that I’m young, and I’m probably confused, but that I can’t go from one boy to another. “Oh, que te crees? Americana? We don’t do things like that (p. 106–7).

The characterization of Gabi as boy crazy (and as a whole) is meant to challenge and unbraid cultural constructs and norms around masculinity.

Quintero does acknowledge the possibility of machista culture’s transformation through the work of men and women. Quintero makes Martin’s masculinity explicitly counter-cultural and conscious of these larger social beliefs of toxic masculinity because Martin’s father is intentionally disrupting “boys will be boys” masculine ideology as well. In talking about his father, Marting says,

He also said that I have to respect you and not pressure you to do things you don’t want to do, and if you say no, it’s no … Yeah. He hates all that macho *boys will be boys* bullshit. He says it’s an excuse for men to act like animals. And I totally agree with him (p. 255).

Through these lines, Quintero offers a new, female-affirmative, responsible, and caring masculine model.

### *Gabi* against the *Lord of the Flies*

These two books present masculinity in competing ways. In *Gabi*, we see the novel explores the cultural, social, and personal aspects of toxic masculinity and, in the process, identifies specific, contemporary structures and beliefs that perpetuate it. Additionally, the novel demonstrates the possibility of a non-toxic masculinity. Masculinity is not necessarily toxic, and men are no less “manly” for supporting women’s choices and wellbeing. *Lord of the Flies* tells a different story, arguing that regardless of culture and society, all men have the beast inside them as part of their very nature. Those who do not allow the beast to manifest in their actions are perceived as feminine, with their boyhood questioned and ostracized.

This difference between both novels can facilitate discussion between adolescent students and their own lived experiences and ideologies. A full, productive discussion on important gender constructs cannot be had without seeing a diversity of claims and portrayals and problems and solutions. *Gabi* offers a narrative of adolescents engaging critically with the world, drawing on their personal experiences as valuable assets, but also critically engaging with systems and ideologies in a contemporary world. *LOF* engages in critical conversations but leaves students with debate on the extent of men’s fated inner beast in his novel, and if this can be extended to Golding’s view of real life. Students should have a broader exploration of how these significant ideas of masculinity, femininity, and social responsibility are understood differently. This is ultimately what critical literacy is, evaluating the implied beliefs and authorship of texts, and how the narratives inform the way we live daily.

*Aristotle and Dante Discover the Secrets of the Universe*

### Ari and Dante: discovering masculinity

*Aristotle and Dante Discover the Secrets of the Universe* follows 15-year-old male protagonist Aristotle Mendoza in his search for meaning, happiness, and answers in his life filled with loneliness, anger, and sadness. This search for meaning and fulfillment takes readers through a course of building up relationships with an emotionally distant and complicated father, coping with nosy friends, resisting intimidation and rivalry with other boys, and making sense of his new friendship with another Mexican-American teen named Dante—a relationship that eventually develops into a romantic one.

Saenz’s dedication in the novel illuminates his authorship and motivation to write the book to speak directly to issues of masculinity and culture. It reads, “To all the boys who have had to learn/to play by different rules” (p. ii). The “different rules” is allusion to the different expressions of masculinity and the challenges men face in their real-life experiences. These lines also recall the moment Dante cries for the death of a bird shot by some boys with a BB gun.

I wanted to tell him not to cry anymore, tell him that what those boys did to that bird didn’t matter. But I knew it did matter. It mattered to Dante. And anyways, it didn’t do any good to tell him not to cry because he needed to cry. That’s the way he was…. And why was it that some guys had tears in them and some had no tears at all? Different boys lived by different rules (Sáenz, 2012, pp. 54-55).

Saenz enters the conversation of masculinity by exploring and validating the experiences of non-traditional boys and men who struggle against toxic masculinity. The scene and dedication represent and affirm the possibility of boys who grapple with wanting to express their masculinity one way while the world holds them to a standard they struggle to live by.

The following section lays out how Saenz explores masculinity and vulnerability through Ari’s search for fulfillment through his relationships, and then explains how this masculine narrative contrasts with key points made by *LOF*. The section also examines how the desert and stars in *A&D* contrast with the island of *LOF*, symbolizing different fulfilled desires of masculinity.

### Ari: a struggle with vulnerability and boyhood

Saenz seems to posit that men, to seek a fulfilling relationship, must allow a significant amount of vulnerability into their lives. This issue of vulnerability is the crux of many characters in the novel. In Ari’s mind, the question of friendship and community is a question of manhood and boyhood. The search for connection is integral to the fulfillment of boyhood and manhood. Ari states:

I was a chair. I felt sadder than I’d ever felt. I knew I wasn’t a boy anymore. But I still felt like a boy. Sort of. But there were other things I was starting to feel. Man things, I guess. Man loneliness was much bigger than boy loneliness (p. 81).

Ari interprets his loneliness as an issue intertwined with his maturation, becoming “a man.” Ari never felt like he could fit in with boys. In discussing other boys who sexually objectify a female pool guard, he states,

…but I always kept my distance from the other boys. I never ever felt like I was a part of their world. Boys. I watched them. Studied them. In the end, I didn’t find most of the guys that surrounded me very interesting. In fact, I was pretty disgusted (p. 22).

As Ari searches for an antidote to his loneliness, he raises legitimate concerns to call into question the community with other boys in his life. Ari’s greatest vulnerability and fear is loneliness, “I wanted to tell her the same thing I wanted to tell Gina Navarro. *Nobody knows me*…. Being on the verge of seventeen could be harsh and painful and confusing” (p. 238).

This struggle for masculine connection is further elaborated in the relationship between Ari and his father, a relationship that is emotionally distant. Both characters are searching for a deeper connection, frustrated by their personal faults and miscommunications. Ari states, “Why could not he just talk? How was I supposed to know him when he did not let me? I hated that” (p. 23). Both Ari and his father had to reveal a emotional trauma to each other before they could begin with the process of healing wounds and seeking connection. Their bad dreams are a common emotional trauma, and once they shared this intimate vulnerability with each other, the relationship became possible. Their dialog about their dreams reveals this deep, intimate yearning for connection:

“You were looking for me,” he said.

I looked at him.

“In your dream. You were looking for me.”

“I’m always looking for you,” I whispered (p. 63).

His father tenderly responds the next day:

“I’m sorry,” he said. “I’m sorry I’m so far away.”

“It’s okay,” I said.

“No,” he said. “No it’s not.”

“I have bad dreams too, Ari.”

All I did was smile at him. He’d told me something about himself. I was happy (p. 66).

The satisfaction of this relationship and this searching between the two begins when they both share this vulnerability with each other. The power of vulnerability is demonstrated again when they both are on a long drive, the two characters reveal current personal and emotional struggles within their relationship and within their self-image:

“I’m sorry about last night,” I said. “It’s just that sometimes I have things running around inside me, these feelings. I don’t always know what to do with them. That probably doesn’t make any sense.”

“It sounds normal, Ari.”

“I don’t think I’m so normal.”

“Feeling is normal.” (p. 280)

In addition to the conversations Ari with his father about his personal life, the new willingness to speak authentically with trust was enough to begin the process of airing out the silence about Bernardo, Ari’s incarcerated brother (p. 283). Additionally, Ari’s father finally began healing from his war-time traumas after revealing this wound more to Ari (p. 347). Therefore, this arc constructs a new, fascinating narrative that boys and men should not have to hide their affections for others. Rather, they should speak freely and process emotional wounds with one another.

### Ari: love and self-love

Saenz also explores the issues of vulnerability, self-love, and masculinity through Ari’s relationship with himself and his relationship with Dante. In Ari’s search for connection and happiness, many barriers were broken until he could understand and acknowledge his romantic feelings for Dante. When they are just getting to know each other, Ari feels comfortable with Dante, “Dante. I really liked him. I really, really liked him” (p. 35). While Ari and Dante both find a sense of joy in their friendship and doing activities together, Dante’s happiness from his personal self-acceptance challenges Ari’s sadness and self-rejection. Ari admires Dante for his self-love but is also challenged by it since Ari cannot love himself, “Until Dante, being with other people was the hardest thing in the world for me. But Dante made talking, living, and feeling seem like all those things were perfectly natural. Not in my world. They were not” (p. 31).

Ari finally finds fulfillment when he begins to understand himself and learns to self-love. After the accident, his helplessness is difficult to accept. Ari states,

I hated that my parents were so patient with me. I did. That’s the truth. They didn’t do anything wrong. They were just trying to help me. But I hated them. And I hated Dante too. And I hated myself for hating them. So there it was, my own vicious cycle. My own private universe of hate (p. 147).

Ari discovers that his self-hate, his fear of acknowledging his wounds is what caused his loneliness. Ari hid, “But I had learned how to hide what I felt. No, that’s not true. There was no learning involved. I had been born knowing how to hide what I felt” (p. 242). It is not until Ari opens his wounds to another person that he can love himself. Once he acknowledges his imperfections, his feelings, emotions, and self-worth.

More importantly, Dante’s acceptance of his romantic feelings for Dante solves the issue of loneliness. His dad finally begins the conversation by saying,

“Ari, it’s time you stopped running … If you keep running, it will kill you.”

“What, Dad?”

“You and Dante.”

“Me and Dante?”

“Ari, the problem isn’t just that Dante’s in love with you. The real problem—for you, anyway—is that you’re in love with him.”

“What am I going to do? I’m so ashamed.”

“Ashamed of what?” my mother said. “Of loving Dante?”

“I’m a guy. He’s a guy. It's not the way things are supposed to be … I hate myself.”

“Don’t, *amor*. *Te adoro*. I’ve already lost a son. I’m not going to lose another…”

“How can you love me so much?”

“How could I not love you? You’re the most beautiful boy in the world.”

“I’m not.”

“You are. *You are*.”

“What am I going to do?”

My father’s voice was soft. “Dante didn’t run. I keep picturing him taking all those blows. But he didn’t run.”

“Okay,” I said. For once in my life, I understood my father perfectly.

And *he* understood *me* (pp. 347–349).

Through the complex relationships between Ari and Dante, Ari and himself, and Ari and his family, Saenz seems to postulate that masculinity requires a degree of vulnerability. Ari’s acceptance of himself and his private feelings was a battle against the traditional cultural norms and expectations around him. His personal struggle was acknowledging his sexuality, which defied traditional conceptions of masculinity. Once he accepted himself through the personal struggle and alongside the loving community that supported him, he felt that freedom he had been searching for in the “secrets of the universe.” The secret, finally understood and accepted, is fulfillment and finally feeling happiness. Ari concludes,

All this time. This was what was wrong with me. All this time I had been trying to figure out the secrets of the universe, the secrets of my own body, of my own heart. All of the answers had always been so close and yet I had always fought them without even knowing it. From the minute I’d met Dante, I had fallen in love with him. I just didn’t let myself know it, think it, feel it…. As Dante and I lay on our backs in the bed of my pickup and gazed out at the summer stars, I was free. Imagine that. Aristotle Mendoza, a free man. I wasn’t afraid anymore (p. 359).

### Masculinity and relationship: conquest or vulnerability?

*A&D* is not the only novel that explores vulnerability and its intersection in masculinity. In *LOF*, masculine relationships are acquired on conquest: boys are only capable of group identification at the expense of individuality, use of violence to solve problems, and conquering each other. The fulfillment of these relationships is solely one-sided, with force and intimidation. The treatment of Piggy by the other boys throughout the book reveals how the boys on the island treat vulnerability. Piggy specifically mentions to Ralph not to call him “Piggy,” yet Ralph takes full advantage of this vulnerability by ridiculing Piggy in front of the older boys to gain more friendship and status from them (p. 29).

A symbolic act of the destruction of vulnerability was the use of war paint. When first used, Golding describes, “[Jack] capered toward Bill, and the mask was a thing on its own, behind which Jack hid, liberated from shame and self-consciousness” (p. 80). The paint served as a way to escape his vulnerabilities and shame. The paint allows Jack to take on a new persona, a persona of brute strength and violence. The war paint treats vulnerability not just as an experience of weakness but judges vulnerability as *the masculine weakness*. From then on, Jack’s relationships and actions became more brutal and violent. Once he finally sets off from the original party and makes his own, this new level of power is clear. He commands the boys to eat the cooked meat, and commanded them to appreciate his work:

Jack spoke again impatiently.

“Has everybody eaten as much as they want?”

His tone conveyed a warning, given out of the pride of ownership, and the boys ate faster while there was still time…. Evening was come, not with calm beauty but with the threat of violence (p. 184).

Vulnerability gets you killed in *LOF*. Not just physical vulnerability, but emotional vulnerability.

*Aristotle and Dante* also depicts the consequences of masculinity without vulnerability; violence and ostracization, both at a societal level and also on a personal level. Dante is a victim of violence because of his non-normative gay orientation (p. 304). This attack obviously highlights the real-life fear and violence that non-normative men face. The novel, though, takes it a step further by highlighting the harm that violence does to Ari himself when he chooses violence.

When Ari responds with similar violence and rage against Dante’s attackers (p. 314). But the anger and violence did not solve anything for Ari, nor for Dante. Anger and violence drive individuals into their “private wars” full of isolation. Ari’s father snaps him out of the violent and angry mindset because of its self-destructive and isolative nature, contrary to the vulnerability and self-love that has led to personal fulfillment. Ar’s father states, “Ari, Ari, Ari. You’re fighting this war in the worst possible way…. You should ask for help” (p. 319).

Ari finally realizes that violence and anger are isolative by nature, “And [I] loved my father too, for the careful way he spoke. I came to understand that my father was a careful man. To be careful with people and with words was a rare and beautiful thing” (p. 324).

In an interesting convergence, *Aristotle and Dante* and *Lord of the Flies* both critique masculinity as callous and brutal. However, where *LOF* emphasizes the abundance of callousness and brutality, *A&D* provides a clearer arc of change and positive modeling of vulnerability in masculine relationships with each other and with themselves for a fulfilling, meaningful existence.

## Conclusion

The interest in and conversations about masculinity will continue to grow, and students will continue to encounter claims and ideologies outside of the classroom. In order to maintain literature instruction relevant to the lived experiences of students, then the classroom must teach students how to critically engage with the culture and the ideologies it carries through its headlines, lyrics, images, and stories (Storey, 2021). When it comes to the topic of masculinity, there are already multiple narratives that contemporary culture reproduces and engages with.

*Ari and Dante* is an example of the personal effects ideologies can have on youth. Ari struggled through unhappiness, self-deprecation, and loneliness. He struggled with self-anger and self-hate. He began to work out his personal suffering when he realized that the pressures around him were forcing him into a box of inauthenticity. In Ari, we can see the personal fulfillment of letting go of these values. There is a clear value in bringing these conversations into the classroom for the benefit of those students who likewise struggle to fit into traditionally defined categories. The YA stories provide a sense of real optimism and hope—a possibility for social and personal change. Literature provides another perspective for students to engage in and reflect on their own actions and how they are engaging with the ideologies in their daily choices. As such, incorporating multiple versions of masculinity through literature assigned and taught in schools could prove beneficial in disrupting adolescent expectations, stereotypes, and ideologies. Future research may consider other versions of masculinity and how they are or are not incorporated into classrooms. We see great potential in researching students’ opinions and interpretations of these contrasting models. We also recognize that we only read across three novels for this study. There are surely many more literary examples—canon and contemporary—that take on masculinity as a construct.

As educators, we often wonder what masculinity narratives our own lives, actions, and behaviors tell in the classroom. But educators can have an active and dialectic relationship with their students. What kind of expectations might we—and other educators—hold our students to? How might our own expectations, language, interactions, rules, and explanations reproduce one narrative over another? How can we challenge the mantra of “boys will be boys” in a place like a school or classroom, where gendered norms and masculinity one-upmanship are often reified and reproduced? The characters we encounter in fiction are characters encountered in real life. Fictional works can construct real-life models of masculinity, and we are optimistic that, despite the recurrence of toxic masculinity in the news, affirmative and positive representations of masculinity can emerge from the page.

## Author contributions

FH: Conceptualization, Writing – original draft. MM: Conceptualization, Writing – original draft, Writing – review & editing.

## References

[ref2] Baker J. R. (1988). Critical essays on William Golding. Boston, Mass: G.K. Hall.

[ref3] Borsheim-Black C. Macaluso M. Petrone R. (2014). Critical literature pedagogy: teaching canonical literature for critical literacy. J. Adolesc. Adult Lit. 58, 123–133. doi: 10.1002/jaal.323

[ref4] Clemens C. (2017). What we mean when we say, “Toxic masculinity.” Learning for Justice. Available at: https://www.learningforjustice.org/magazine/what-we-mean-when-we-say-toxic-masculinity.

[ref5] Connell R. W. (2005). Masculinities. London: Routledge.

[ref6] De Boise S. (2019). Is masculinity toxic? Norma 14, 147–151. doi: 10.1080/18902138.2019.1654742, PMID: 38012906

[ref8] Golding W. (1962). Lord of the flies: Education. Faber and Faber Limited. London, England.

[ref9] Krippendorff K. (2004). Content analysis: An introduction to its methodology (2nd ed.) Thousand Oaks, CA: Sage Publications.

[ref9001] Macaluso M. (2016). Examining canonicity as an implicit and discursive frame in secondary English classrooms (Doctoral dissertation). Available at: https://d.lib.msu.edu/etd/4079

[ref10] Macaluso M. (2017). Teaching to kill a mockingbird today: coming to terms with race, racism, and America’s novel. J. Adolesc. Adult Lit. 61, 279–287. doi: 10.1002/jaal.678

[ref11] Quintero I. (2014). Gabi, A Girl in Pieces. El Paso: Cinco Punto Press.

[ref12] Sáenz B. A. (2012). Aristotle and Dante discover the secrets of the universe. New York: Simon & Schuster

[ref13] Steiss J. (2020). Dismantling winning stories: lessons from applying critical literature pedagogy to the odyssey. J. Adolesc. Adult Lit. 63, 433–441. doi: 10.1002/jaal.1012

[ref14] Storey J. (2021). Cultural theory and popular culture: an introduction. London: Routledge.

